# Effects of Dietary Fiber and Its Components on Metabolic Health

**DOI:** 10.3390/nu2121266

**Published:** 2010-12-15

**Authors:** James M. Lattimer, Mark D. Haub

**Affiliations:** Department of Human Nutrition, Kansas State University, 127 Justin Hall, Manhattan, KS 66506, USA; Email: jml3468@k-state.edu

**Keywords:** fiber, obesity, diabetes, cardiovascular, arabinoxylan, inulin, pectin, bran, cellulose, β-glucan resistant starch

## Abstract

Dietary fiber and whole grains contain a unique blend of bioactive components including resistant starches, vitamins, minerals, phytochemicals and antioxidants. As a result, research regarding their potential health benefits has received considerable attention in the last several decades. Epidemiological and clinical studies demonstrate that intake of dietary fiber and whole grain is inversely related to obesity, type two diabetes, cancer and cardiovascular disease (CVD). Defining dietary fiber is a divergent process and is dependent on both nutrition and analytical concepts. The most common and accepted definition is based on nutritional physiology. Generally speaking, dietary fiber is the edible parts of plants, or similar carbohydrates, that are resistant to digestion and absorption in the small intestine. Dietary fiber can be separated into many different fractions. Recent research has begun to isolate these components and determine if increasing their levels in a diet is beneficial to human health. These fractions include arabinoxylan, inulin, pectin, bran, cellulose, β-glucan and resistant starch. The study of these components may give us a better understanding of how and why dietary fiber may decrease the risk for certain diseases. The mechanisms behind the reported effects of dietary fiber on metabolic health are not well established. It is speculated to be a result of changes in intestinal viscosity, nutrient absorption, rate of passage, production of short chain fatty acids and production of gut hormones. Given the inconsistencies reported between studies this review will examine the most up to date data concerning dietary fiber and its effects on metabolic health.

## 1. Introduction

Dietary fiber and whole grains contain a unique blend of bioactive components including resistant starches, vitamins, minerals, phytochemicals and antioxidants. As a result, research regarding their potential health benefits has received considerable attention in the last several decades. Epidemiological and clinical studies demonstrate that consumption of dietary fiber and whole grain intake is inversely related to obesity [1], type two diabetes [2], cancer [3] and cardiovascular disease (CVD) [4].

The Food and Drug Administration (FDA) has approved two health claims for dietary fiber. The first claim states that, along with a decreased consumption of fats (<30% of calories), an increased consumption of dietary fiber from fruits, vegetables and whole grains may reduce some types of cancer [5]. “Increased consumption” is defined as six or more one ounce equivalents, with three ounces derived from whole grains. A one ounce equivalent would be consistent with one slice of bread, ½ cup oatmeal or rice, or five to seven crackers. 

Recent studies support this inverse relationship between dietary fiber and the development of several types of cancers including colorectal, small intestine, oral, larynx and breast [3,6,7]. Although most studies agree with these findings, the mechanisms responsible are still unclear. Several modes of actions however have been proposed. First, dietary fiber (DF) resists digestion in the small intestine, thereby allowing it to enter the large intestine where it is fermented to produce short chain fatty acids, which have anti-carcinogenic properties [8]. Second, since DF increases fecal bulking and viscosity, there is less contact time between potential carcinogens and mucosal cells. Third, DF increases the binding between bile acids and carcinogens. Fourth, increased intake of dietary fiber yield increased levels of antioxidants. Fifth, DF may increase the amount of estrogen excreted in the feces due to an inhibition of estrogen absorption in the intestines [9]. 

The second FDA claim supporting health benefits of DF states that diets low in saturated fat (<10% of calories) and cholesterol and high in fruits, vegetables and whole grain, have a decreased risk of leading to coronary heart disease (CHD) [10]. For most, an increased consumption of dietary fiber is considered to be approximately 25 to 35 g/d, of which 6 g are soluble fiber. 

Obviously, many studies support the inverse relationship of dietary fiber and the risk for CHD. However, more recent studies found interesting data illustrating that for every 10 g of additional fiber added to a diet the mortality risk of CHD decreased by 17–35% [11,4]. Risk factors for CHD include hypercholesterolemia, hypertension, obesity and type two diabetes. It is speculated that the control and treatment of these risk factors underlie the mechanisms behind DF and CHD prevention. First, soluble fibers have been shown to increase the rate of bile excretion therefore reducing serum total and LDL cholesterol [12]. Second, short chain fatty acid production, specifically propionate, has been shown to inhibit cholesterol synthesis [13]. Third, dietary fiber demonstrates the ability to regulate energy intake thus enhancing weight loss or maintenance of a healthier body weight. Fourth, either through glycemic control or reduced energy intake, dietary fiber has been shown to lower the risk for type two diabetes. Fifth, DF has been shown to decrease pro-inflammatory cytokines such as interleukin-18 which may have an effect on plaque stability [14]. Sixth, increasing DF intake has been show to decrease circulating levels of C-Reactive protein (CRP), a marker of inflammation and a predictor for CHD [15].

Although only two claims have been adopted and supported by the FDA, multiple other “potential claims” have been researched and well documented. Those conditions of particular importance, due to their increasing prevalence among the general population, include obesity and type two diabetes [16,17]. The digestive physiology of dietary fiber has significant implications in the risk for, and treatment of, these metabolic disorders. Dietary fibers have been shown to result in decreased blood glucose excursions and attenuated insulin responses. This may be due to either a delayed [18] or decreased [1] intestinal absorption. Therefore, the purpose of the paper is to review current research regarding dietary fiber and whole grains in relation to these proposed claims. Focus will be placed on nutrient absorption, postprandial glycemia, insulin sensitivity, caloric density and satiety. Furthermore, select constituents of DF will be discussed in detail to better define their role in metabolic health. 

## 2. Defining Dietary Fiber

In the simplest form, carbohydrates can be separated into two basic groups based upon their digestibility in the GI tract. The first group (*i.e.*, starch, simple sugars, and fructans) is easily hydrolyzed by enzymatic reactions and absorbed in the small intestine. These compounds can be referred to as non-structural carbohydrates, non-fibrous polysaccharides (NFC) or simple carbohydrates. The second group (*i.e.*, cellulose, hemicellulose, lignin, pectin and beta-glucans) are resistant to digestion in the small intestine and require bacterial fermentation located in the large intestine. These compounds can be referred to as complex carbohydrates, non-starch polysaccharide (NSP) or structural carbohydrates and are reflective in Neutral Detergent Fiber (NDF) and Acid Detergent Fiber (ADF) analysis. NDF consists of cellulose, hemicelluloses and lignin while ADF consists of cellulose and lignin. However, NDF and ADF analysis are used primarily for animal nutrition and the analysis of roughages. 

Although, NDF and ADF are typically not used in human nutrition, the separation of structural (NSP) and non-structural carbohydrates provide the basis for beginning to define and understand dietary fiber. This task has been a divergent process and has depended on both nutrition and analytical concepts. The most common and accepted definition is based on nutritional physiology. However, chemists and regulatory boards have leaned toward analytical procedures to factually define dietary fiber. The physiological definition is easier for the general public to understand and adopt for practical use. 

### 2.1. American Association of Cereal Chemists

A recent description, as suggested by the American Association of Cereal Chemists [19], terms dietary fiber as carbohydrate polymers with more than a three degree polymerization which are neither digested nor absorbed in the small intestine. The greater than three degree polymer rule was designed to exclude mono and disaccharides. The known constituents of dietary fiber are listed in Table 1. 

This definition describes in more detail the components of dietary fiber as well as its genetic makeup. Furthermore, the changes set forth in its description require few changes for its analytical evaluation [20].

### 2.2. World Health Organization and Food and Agriculture Organization

The World Health Organization (WHO) and Food and Agriculture Organization (FAO) agree with the American Association of Cereal Chemists (AACC) definition but with a slight variation. They state that dietary fiber is a polysaccharide with ten or more monomeric units which is not hydrolyzed by endogenous hormones in the small intestine [21].

**Table 1:** Components of dietary fiber according to the American Association of Cereal Chemists [22].

**Non Starch Polysaccharides and Oligosaccharides**	
	Cellulose
	Hemicellulose
	Arabinoxylans
	Arabinogalactans
	Polyfructoses
	Inulin
	Oligofructans
	Galacto-oligosaccharides
	Gums
	Mucilages
	Pectins
**Analagous carbohydrates**	
	Indigestible dextrins
	Resistant maltodextrins
	Resistant potato dextrins
	Synthesized carbohydrates compounds
	Polydextrose
	Methyl cellulose
	Hydroxypropylmethyl cellulose
	Resistant starches
**Lignin substances associated with the NSP and lignin complex**	
	Waxes
	Phytate
	Cutin
	Saponins
	Suberin
	Tannin

### 2.3. Soluble *versus* Insoluble

NSP can be further subdivided into the two general groups of soluble and insoluble. This grouping is based on chemical, physical, and functional properties [23]. Soluble fiber dissolves in water forming viscous gels. They bypass the digestion of the small intestine and are easily fermented by the microflora of the large intestine. They consist of pectins, gums, inulin-type fructans and some hemicelluloses. In the human GI tract, insoluble fibers are not water soluble. They do not form gels due to their water insolubility and fermentation is severally limited. Some examples of insoluble fiber are of lignin, cellulose and some hemicelluloses. Most fiber containing foods include approximately one-third soluble and two-third insoluble fiber [24].

## 3. Proposed Health Benefits of Dietary Fiber

Dietary fiber and whole grains are an abundant source of nutrients including vitamins, minerals, and a slowly digestible energy. In addition, they contain phytochemicals such as phenolics, carotenoids, lignans, beta-glucan and inulin. These chemicals, secreted by plants, are not currently classified as essential nutrients but may be important factors in human health [25]. The synergistic effect of phytochemicals, increased nutrient content and digestive properties, are believed to be the mechanism behind dietary fibers beneficial effects on the treatment and prevention of obesity and diabetes [1,26], reduced CVD [27] and decreased incidence of certain types of cancer [28,29]. In the following subsections, potential health benefits of dietary fiber will be reviewed along with their possible mechanisms and modes of actions.

### 3.1. Obesity

Approximately 66% of U.S. adults are overweight or obese [16] resulting in an increased risk of health problems including, but not limited to, diabetes, CVD, and certain types of cancer [30]. Although there are multiple factors that could contribute to obesity, the primary cause is due to an increase in the energy absorption:energy expenditure ratio. Therefore, limiting energy absorption is critical when treating obesity. Scientists have taken this a step further and studied the effect of other dietary aspects that may serve in weight regulation, including dietary fiber. Increasing dietary fiber consumption may decrease energy absorption by way of diluting a diet’s energy availability while maintaining other important nutrients. 

Substantial research has been conducted to evaluate the effect of dietary fiber and body weight, most all of which show an inverse relationship between dietary fiber intake and change in body weight. Tucker and Thomas [1] supported this statement in a study consisting of 252 middle aged women. They observed that over a 20 month period participants lost an average of 4.4 lbs due to an 8 g increase in dietary fiber per 1000 kcal. This weight loss was primarily due to decreased body fat. It should be recognized that the correlation between dietary fiber and weight change was independent of many other potential factors including age, baseline fiber and fat intakes, activity level, and baseline energy intake. 

Koh-Banerjee *et al.* [31] concur with the above findings and also suggest a dose-response relationship. They reported that for every 40 g/d increase in whole grain intake, weight gain decreased by 1.1 lbs. Moreover, bran seemed to play an important role in the reduction of weight gain by 0.8 lbs per 20 g/d intake. 

Dietary fiber’s ability to decrease body weight or attenuate weight gain could be contributed to several factors. First, soluble fiber, when fermented in the large intestine, produces glucagon-like peptide (GLP-1) and peptide YY (PYY) [32]. These two gut hormones play a role inducing satiety. Second, dietary fiber may significantly decrease energy intake [1]. Women who consumed increased levels of fiber tended to also have a decreased consumption of dietary fat. Third, dietary fiber may decrease a diets metabolizable energy (ME), which is gross energy minus the energy lost in the feces, urine and combustible gases. Baer *et al.* [33] observed that an increased consumption of dietary fiber resulted in a decrease in the ME of the diet. This may be attributed to the fact that fat digestibility decreased as dietary fiber increased. Also, as dietary fiber intake increases, the intake of simple carbohydrates tends to decrease. Although, dietary fiber still contributes to the total caloric content of a diet, it is much more resistant to digestion by the small intestine and even somewhat resistant in the large intestine. 

It should also be noted that the inverse relationship between dietary fiber and ME was independent of dietary fat. Therefore, ME decreased as dietary fiber increased in both high and low fat diets. However, when dietary fiber was split into soluble and insoluble fiber, the results were much more inconclusive. Soluble fiber decreased ME when added to a low fat diet but increased ME when added to a high fat diet [33]. It is not really known how dietary fat changes the effects of soluble fiber. Isken *et al.* [34] showed supportive data in mice consuming a high fat diet. Mice showed an increased weight gain when soluble fiber was added to a high fat diet. There are several mechanisms that may explain how soluble fiber could increase ME or weight gain. First, bacterial populations in the large intestine increase due to an increase in soluble fiber consumption [35]. This could result in increased fermentation and utilization of short chain fatty acids thereby increasing energy absorption. Second, soluble fiber enlarges in the GI and forms a viscous material which delays intestinal transit time [36]. Subsequently, this increase time in the GI tract may allow for more complete digestion and absorption. Conversely, some believe this increase viscosity has an opposite effect and retards absorption [26]. More research is needed in this area.

Insoluble fiber seems to have the opposite effect to that of soluble. When insoluble fiber intake was increased in mice consuming a high fat diet, body weight decreased [34]. Research in sows demonstrated that insoluble fiber decreased energy digestibility while it increased with soluble fiber intake [37]. The mode of action behind these findings may be due to the fact that insoluble fiber causes an increased rate of passage through the GI tract [38]. This would be expected to result in diminished digestion and absorption of nutrients. 

According to the data presented above, both soluble and insoluble fiber may lead to weight loss. However, there seems to be a relationship between the type of diet (high or low fat) and the type of fiber consumed. Insoluble fiber may play a more important role for weight loss during consumption of a high fat diet. Since resistant starch is a constituent of dietary fiber and undergoes the same digestion as insoluble fiber, comparing resistant starch and insoluble fiber may give us a better understanding of how dietary fiber can be used to treat and prevent obesity. Adding resistant starch to a diet dilutes its ME, but not to the degree of insoluble fiber [39].

Numerous studies [31,40] have found the same inverse relationship between dietary fiber and weight gain. However, the data are more inconsistent when comparing soluble and insoluble. Thus, although increasing dietary fiber in general has a favorable effect on body weight, more research is warranted to determine the optimal dietary fibers for the purpose of weight management. 

### 3.2. Diabetes

Type two diabetes has increased exponentially over the past several years. Since 1990, self reported diabetes increased 61% [17]. Although other risk factors such as obesity, lack of physical activity and smoking are precursors for the disease, dietary factors also seem to play a significant role. Type two diabetes results from decreased insulin sensitivity and hyperglycemia. For that reason, a primary dietary factor of particular concern is carbohydrate intake. 

Meyer *et al.* [41] observed that total carbohydrates had no effect on the risk of diabetes. However, the type of carbohydrate (non structural carbohydrates and dietary fiber) was significantly related. Therefore, it is important to understand a foods glycemic index or load. Glycemic index ranks total carbohydrate intake according to their immediate postprandial glucose response in comparison to a reference group such as glucose or white bread. Carbohydrates with a low glycemic index result in a smaller glucose/insulin response. Simple small chain carbohydrates would be considered to have a higher glycemic index since it produces higher blood glucose concentrations. 

While Hu *et al.* [42] found that excess body fat was the single most important determinant of type two diabetes, poor nutrition was also a large influential factor. Moreover, women consuming a poor diet significantly increased their risk of developing type two diabetes. Poor diet was classified as a diet high in saturated fat, low dietary fiber and high non structural carbohydrates. This diet would be consistent with a high glycemic load being higher in easily digestible and rapidly absorbable carbohydrates. In a supportive, long term (eight year) study of over 90,000 female nurses, Shuzle *et al.* [43] found a positive correlation between glycemic index and risk of type two diabetes. This association was significant even after adjusting for age, body mass index (BMI) and family history. Several means have been proposed to understand the physiology behind the relationship of glycemic index and diabetes. First, carbohydrates with a higher glycemic index produce higher blood glucose levels. This chronic hyperglycemia is suggested to lead to the dysfunction of beta cells in the pancreas thus decreasing insulin release. Second, due to an over abundance of energy (*i.e.*, high glycemic load) tissues such as skeletal muscle, liver and adipose become resistant to insulin. 

Although a majority of studies show a positive correlation between high glycemic foods and type two diabetes, several studies disagree with these findings. Meyer *et al.* [41] found that glycemic index had no effect on the prevalence of diabetes in older aged women. However, there was a strong (*P* = 0.005) inverse relationship between dietary fiber intake and diabetes when adjusted for age and BMI. Women consuming an average of 26 g/d of dietary fiber had a 22% lower risk of developing diabetes when compared to women only consuming 13 g/d. Schulze *et al.* [43] agreed with these findings with men and women showing a decreased risk of diabetes (*P* < 0.001) with the consumption of an additional 12 g of dietary fiber per day. According to these findings, it may be more significant to focus on an increased consumption of dietary fiber to prevent diabetes than glycemic index/load. 

It is important to note that the inverse relationship between dietary fiber and diabetes observed by Meyer *et al.* [41] and Schulze *et al.* [43] was independent of age and body weight. Hu *et al.* [42] supported these findings while correcting for age, fat intake, smoking, alcohol, family history, exercise, and body weight. Therefore, it seems that dietary fiber is associated with type two diabetes, independent of other compounding factors.

According to recent research, the soluble *versus* insoluble fraction of fiber may give some insight on the efficacy of dietary fiber on diabetes and its mechanisms. Early research regarding soluble fiber demonstrated delayed gastric emptying and decreased absorption of macronutrients, resulting in lower postprandial blood glucose and insulin levels [44]. This is most likely due to the viscosity of soluble fibers inside the GI tract. Interestingly, different types of soluble fiber had varying effects on viscosity and nutrient absorption. Guar had the highest viscosity as well as the greatest effect at decreasing postprandial blood glucose. Therefore, it would be assumed that an increased level of soluble fiber would be associated with a decreased risk of diabetes. However, several recent studies have demonstrated the opposite showing no correlation between soluble fiber and a reduced risk of diabetes [41,43,45].

Although some studies have been contradictory, showing no differentiation between soluble and insoluble fiber on diabetes [43], a majority of the research demonstrates a strong inverse relationship between insoluble fiber and the risk of type two diabetes. Meyer *et al.* [41] using healthy middle aged women, observed a strong (*P* = 0.0012) inverse relationship between insoluble fiber and the risk of type two diabetes while soluble fiber had no effect. Montonen *et al.* [45] also found the same results in healthy middle aged men and women consuming increased levels of whole rye bread. Interestingly, fiber from fruits and vegetables had no effect on the risk of developing type two diabetes. Earlier studies have agreed with these findings. A large epidemiological study of 42,000 men found that dietary fiber from fruits or vegetables had no effect on the risk of diabetes. However, dietary fiber from whole cereal grains showed a significant decrease in diabetes occurrence [46]. Daily intakes of fiber among all groups were similar. 

Insoluble fiber only has a small effect on macronutrient absorption [44]. Therefore, another mode of action must be present and several hypotheses should be discussed. Some suggest that insoluble fiber increases the passage rate of foodstuff through the GI tract thus resulting in a decreased absorption of nutrients, namely simple carbohydrates. However, Weicket *et al.* [26] found that an increased intake of cereal fiber significantly improved whole body glucose disposal resulting in an 8% improvement of insulin sensitivity. This suggests that the mechanisms behind insoluble fiber are more peripheral and not limited to nutrient absorption. First, an accelerated secretion of glucose-dependent insulintropic polypeptide (GIP) was observed directly after the ingestion of an insoluble fiber in healthy women [47]. GIP is an incretin hormone which stimulates postprandial insulin release. Second, insoluble fiber can result in a reduced appetite and food intake [48]. This may lead to a decreased caloric intake and BMI as described in the obesity section of this review. Third, short chain fatty acids, via fermentation, have been shown to reduce postpandrial glucose response [49,50]. Early research demonstrated that lipid infusions impaired glucose utilization [51] and oral acetate could decrease free fatty acids (FFA) in the blood [52]. According to Kelley and Mandarino [53], increases in FFA in the blood can inhibit glucose metabolism through the inhibition of GLUT 4 transporters. Therefore, short chain fatty acids, by way of decreasing serum free fatty acids, may reduce blood glucose levels through competition in insulin–sensitive tissues. 

The inverse relationship between cereal grains and diabetes may also be attributed to an increased consumption of magnesium. Increased intake of magnesium has been shown to decrease the incidence of type two diabetes [41,54]. Hypomagnesemia is common among diabetics and has been associated with a reduction of tyrosine kinase at the insulin receptor [55]. This may impair the action of insulin thus leading to insulin resistance. 

According to recent research, increasing levels of dietary fiber may contribute a non–pharmacological way to improve carbohydrate metabolism. However, some inconsistency does exist and may be contributed to the classification of dietary fiber and whole grains. Subjects with diagnosed type two diabetes may also add to the irregularity among the data. In a study of men and women with established diabetes, Jenkins *et al.* [56] observed that wheat bran had no effect on glycemic control in subjects with type two diabetes. Much of the research showing improved glycemia with dietary fiber examined healthy subjects, rather than subjects with diagnosed type two diabetes. Further research is needed to determine whether dietary fiber may be able to aid in the control of normal blood glucose levels in subjects with established type two diabetes. 

## 4. Fiber Components of Interest

Dietary fiber can be separated into many different factions (Table 1). Recent research has begun to isolate these components and determine if increasing their levels in a diet is beneficial to human health. The separation of these fractions may give us a better understanding of how and why dietary fiber may decrease the risk for certain diseases.

### 4.1. Arabinoxylan

Arabinoxylan (AX), a constituent of hemicelluloses, is comprised of a xylose backbone with arabinose side chains. AX is a major component of dietary fiber in whole grains having considerable inclusions in both the endosperm and bran. In wheat, AX account for around 64–69% of the NSP in the bran and around 88% in the endosperm [57]. During normal wheat flour processing, a majority of the AX is removed as a by-product. In the GI tract, AX acts much like a soluble fiber being rapidly fermented by the microflora of the colon. 

Lu *et al.* [58], observed an inverse relationship between the intake level of an AX rich bread and postprandial glucose response in healthy adult subjects. When compared to the control, postprandial glucose levels were significantly lower with only 6 g of AX rich fiber supplementation while 12 g produced the greatest benefit. Breads high in AX also appear to control blood glucose and insulin in adults with an already impaired glucose tolerance [59]. Fasting blood glucose, postprandial blood glucose and insulin were all significantly lower when adults with type two diabetes were supplemented with 15 g/d of an AX rich fiber. The mode of action behind AX on improving glucose tolerance is unknown. However, it is thought to be due to the high viscosity of the fiber inside the lumen of the GI tract, thereby slowing the rate of glucose absorption.

The lower glycemic index of AX may also play a role. Breads made with a flour rich in AX have a relatively low glycemic index of around 59. Whole wheat flour, although high in fiber, has a glycemic index of around 99 [58]. Arabinoxylan rich bread has a similar glycemic index to that of whole grain bread but offers some distinct advantages such as improved mouth feel and tenderness. There was no significant difference in the sensory analysis between the control and a bread containing 14% AX rich fiber [58]. 

### 4.2. Inulin

Inulin is a polymer of fructose monomers and is present in such foods as onions, garlic, wheat, artichokes and bananas and is used to improve taste and mouthfeel in certain applications. It is also used as a functional food ingredient due to its nutritional properties. Because of this, inulin products can be used as a replacement for fat or soluble carbohydrates without affecting the taste and texture and still contribute to a foods nutritional value. 

Enzymatic hydrolyses in the small intestine is minimal (<10%) since inulin consists of beta bonds. Therefore, it enters the large intestine and is almost completely metabolized by the microflora. When fermented, they tend to favor propionate production which, in turn, decreases the acetate to propionate ratio leading to decreased total serum cholesterol and LDL [13], which are important risk factors for CHD. 

Inulin has also demonstrated the ability to contribute to the health of the human large intestine as a prebiotic [60]. They demonstrated that inulin stimulated the growth of *bifidobacteria* while restricting the growth of potential pathogenic bacteria such as *E. coli*, *Salmonella*, and *Listeria*. This could prove to be beneficial in such disorders as ulcerative colitis and *C. difficile* infections. Rafter *et al.* [61] agreed with these findings and suggested they were the underlying mechanisms behind the observation that inulin decreased biological compounds associated with colonic cancer, including reduced colorectal cell proliferation and water induced necrosis, decreased exposure to genotoxins, and decreased interleukin-2 release. 

Increased mineral absorption may also contribute to the functionality of inulin. Increased calcium absorption, by approximately 20%, was reported in adolescent girls supplemented with inulin [62]. Results from Abrams *et al.* [63], support these findings in a longer (one year) study of pubertal boys and girls consuming an inulin supplement. Subjects in the treatment group also experienced increased bone mineral density when compared to the control. The mechanisms behind these findings are still unclear but may be due to increased calcium absorption from the colon or possibly an increased solubility in the lumen of the GI tract due to short chain fatty acids. Finally, it may increase absorption through an enhancement of vitamin D. 

Inulin may also provide a way to prevent and treat obesity. Cani *et al.* [64] demonstrated that oligofructose, a subgroup of inulin, increased satiety in adults which led to a decrease in total energy intake. This is thought to be due to short chain fatty acids and their ability to increase appetite suppressing hormones such as glucagon-like peptide 1 (GLP-1). 

### 4.3. β-glucan

β-glucan is a linear polysaccharide of glucose monomers with β(1→4) and β(1→3) linkages and found in the endosperm of cereal grains, primarily barley and oats. β-glucan concentrations in North American oat cultivars range from 3.9% to 6.8% [65]. β-glucan is water soluble and highly viscous at low concentrations [66].

The physiological benefits due to β-glucan seem to stem from their effect on lipid metabolism and postprandial glucose metabolism. Many studies agree an inverse relationship exists between consumption levels of β-glucan and cholesterol levels. Several recent studies, in both hypercholesterolemic [67] and healthy [68] subjects, found that the daily consumption of 5 g of β–glucan significantly decreased serum total and LDL cholesterol. Davidson *et al.* [35] found that only a daily consumption of 3.6 g β-glucan was needed to produce the same significant effects. The same relationship also has been reported to occur between β-glucan and postprandial glucose and insulin responses in both diabetic and healthy subjects. Biorklund *et al.* [69] found that 5 g of β-glucan from oats significantly decreased postprandial glucose and insulin levels in healthy adults. Tappy *et al.* [70] reported the same results in adult subjects diagnosed with type two diabetes who consumed 4.0, 6.0 or 8.4 g of β-glucan. 

Most authors agree that β-glucan’s viscosity in the GI tract is the most probable mechanism in which it decreases serum cholesterol levels as well as improves post prandial glucose metabolism. This gellation property may decrease bile acid absorption by increasing intestinal viscosity and increase bile acid excretion. This subsequently results in a higher hepatic cholesterol synthesis because of the higher need for bile acid synthesis [71]. The same viscosity may also delay glucose absorption into the blood thus lowering post prandial glucose and insulin levels. Nazare *et al.* [72] observed that 5 g of oat β–glucan added to an oat concentrate cereal significantly delayed, but did not reduce, total glucose absorption. 

The production of short chain fatty acids from β-glucan may also be a probable mechanism behind its observed metabolic effects. Fermentation of oat β-glucan has been shown to yield larger amounts of propionate [73,74]. Propionate has been shown to significantly inhibit cholesterol synthesis in humans [13] and is thought to be due to the inhibition of the rate limiting enzyme HMG CoA reductase [75]. 

Not all research however, agrees that β-glucan can affect lipid and glucose absorption/metabolism. Keogh *et al.* [76] observed that treatments of 8.1 to 11.9 g/d of barley β-glucan had no effect on total or LDL cholesterol in mildly hyperlipidemic adults. Cugent-Anceau *et al.* [77] not only observed that 3.5 g of oat β-glucan added to soup did not alter serum lipid profiles, but also produced no change in postprandial glucose levels. 

The inconsistency between studies is thought to be due to the molecular weight (MW) and solubility of the β-glucan. The MW can be changed by several factors including food processing and the source of the β-glucan. Suortti *et al.* [78] states that heating, such as in extrusion and baking, decreases the molecular weight of β-glucan therefore decreasing its viscosity inside the GI tract. Theuwissen and Meinsk [67] and Naumann *et al.* [68] both used a β-glucan obtained through a dry milling process in which it is not significantly degraded. Keogh *et al.* [78] however, obtained their β–glucan from a hot water extraction process which may have decreased MW and in turn intestinal viscosity. Kerckhoffs *et al.* [79] supports this theory as they observed that β-glucan, when added to bread or cookies, produced no change in the lipoprotein profile of mildly hypercholesterolemic adults. However, when the same β-glucan was added to orange juice, serum LDL decreased significantly. The bread baking process decreased the MW of the β-glucan. Unfortunately, this study failed to list the MW weight of the β-glucan in the orange juice. 

Different sources of β-glucan may also differ in their molecular weight and viscosity. Oats were the β-glucan source for the Theuwissen and Meinsk [67] and Naumann *et al.* [68] studies while Keogh *et al.* [76] utilized barley. Biorklund *et al.* [69] found similar results in that 5 g of β-glucan derived from oats significantly lowered serum cholesterol and postprandial glucose and insulin levels while the same level of β-glucan derived from barley produced no effects. Since the MW can vary among oat varieties [80] it may be safe to assume that the MW can also vary among cereal grain types. 

Oat and barley β-glucan also seem to differ in their solubility which would have a direct effect on intestinal viscosity. Gaidosova *et al.* [81] found that the solubility of barely β-glucan was significantly higher than that of oats. 

Oat and barley varieties may also play a role on the MW of β-glucan. Yao *et al.* [80] observed large differences in viscosity between β-glucan solutions from different oat varieties as a result of β-glucan’s wide range of molecular weights. Torronen *et al.* [82] using a lower MW (370,000 da) β-glucan found no changes in serum lipid profiles in men with mild to moderate hypercholesterolaemia when compared to a control. Cugent-Anceau *et al.* [83], also using a low molecular weight (80,000 da) β–glucan, found similar negative results. However, when a high MW (1,200,000 da) β-glucan was used, it reduced serum cholesterol levels in the same class of subjects [84]. Kim *et al.* [73] disagreed with these findings when they reported that a lower molecular weight β-glucan bound significantly more bile acid *in vitro*. It should be noted however, that the β-glucan in this study was not in its natural form. Extracted β-glucan was treated with lichenase to yield the different molecular weights. 

### 4.4. Pectin

Pectin is a linear polymer of galacturonic acid connected with α (1→4) bonds. Regions of this backbone are substituted with α (1→2) rhamnopyranose units from which side chains of neutral sugars such as galactose, mannose, glucose and xylose occur. Pectin is a water soluble polysaccharide that bypasses enzymatic digestion of the small intestine but is easily degraded by the microflora of the colon. Citrus fruit contains anywhere from 0.5% to 3.5% pectin with a large concentration located in the peel. Commercially extracted pectins are also available and are typically used in food applications which require a gelling or a thickening agent. 

Inside the GI tract, pectin maintains this ability to form a gel or thicken a solution. This is thought to be the likely mechanism behind its many beneficial effects on health including dumping syndrome [85], improved cholesterol and lipid metabolism [86], and diabetes prevention and control [87]. However, pectin also contains some unique abilities that may treat or prevent other diseases/disorders such as intestinal infections, atherosclerosis, cancer and obesity. 

Several recent clinical studies, Rabbani *et al.* [88] and Triplehorn and Millard [89], demonstrated that oral pectin supplementation to children and infants reduced acute intestinal infections and significantly slowed diarrhea. This is thought to be due to a reduction in pathogenic bacteria such as *Shigella*, *Salmonella*, *Klebsiella*, *Enterobacter*, *Proteus* and *Citrobacter*. This is supported by Olano–Martin *et al.* [90] who observed that pectin stimulated the growth of certain strains of *Bifidobacteria* and *Lactobacillus in vitro*. These bacteria are considered to be directly related to the health of the large intestine and their concentrations depict a healthy microflora population. 

The quality of fibrin is thought to be an important risk factor for atherosclerosis, stroke and coronary heart disease. Pectin has been shown to increase fibrin permeability and decrease fibrin tensile strength in hyperlipidaemic men [91]. Although the mechanism behind this is unknown, it is thought to be due in part to acetate production. Pectin yields predominantly acetate in the colon which is thought to enter peripheral circulation and alter fibrin architecture. 

Pectin may also have a potential role in the complicated area of cancer prevention. Nangia–Makker *et al.* [92] found that pectin was able to bind to and decrease tumor growth and cancerous cell migration in rats fed modified citrus pectin. This is thought to be a result of pectin binding to galectin-3 and inhibiting some of its functions.

### 4.5. Bran

Bran is the outer most layer of a cereal grain and consists of the nucellar epidermis, seed coat, pericarp and aleurone. The aleurone consists of heavy walled, cube shaped cells which are composed primarily of cellulose. It is low in starch and high in minerals, protein, and fat. However, due to its thick cellulosic walls, these nutrients are virtually unavailable for digestion in monogastric species. The AACC defines oat bran as “*the food which is produced by grinding clean oat groats or rolled oats and separating the resulting oat flour by sieving bolting, and/or other suitable means into fractions such that the oat bran fraction is not more than 50% of the original starting material and has a total betaglucan content of at least 5.5% (dry-weight basis) and a total dietary fiber content of at least 16.0% (dry-weight basis), and such that at least one-third of the total dietary fiber is soluble fiber.*” [19].

Bran from a wide array of cereal grains have been shown to have an effect on postprandial glucose levels, serum cholesterol, colon cancer, and body mass. Although the efficacy of bran may change due to its source, the purpose of this section will just evaluate bran’s general effect on the parameters listed above. 

In a recent study of healthy adults, 31 g of rye bran decreased peak postprandial glucose levels by 35% when compared to the control [93]. This effect may be due to the high AX content in rye bran. Arabinoxylan, as discussed previously, may increase intestinal viscosity and slow nutrient absorption. In a more lengthy study, Qureshi *et al.* [94] found that subjects suffering type one and two diabetes decreased their fasting glucose levels due to the daily consumption of 10 g of stabilized rice bran over two months. The results may arise due to an increased intestinal viscosity, but is more likely a result of a decreased carbohydrate/caloric intake. Koh-Banerjee *et al.* [31], in a larger clinical study, supports this theory in their finding that for every 20 g/d increase in consumption of bran, body weight decreased by 0.80 lbs. It should be noted that this data remained significant even after adjustment for fat and protein intake, daily activity, caloric intake and baseline weight. In an earlier study, Zhang *et al.* [95] observed that adults with ileostomies, consuming bread rich in rye bran, significantly increased the ileal excretion of fat, nitrogen and energy. This study suggests bran did not delay nutrient absorption in the small intestine but hindered it.

In addition to a possible effect on carbohydrate absorption and metabolism, bran also seems to have the same effect on lipids. In a long term clinical study, Jensen *et al.* [96] reported that an increased daily consumption of bran significantly decreased the risk of coronary heart disease in healthy adult men. This is most likely due to the data reported by Qureshi *et al.* [94] who found that 10 g of rice bran consumed for eight weeks was able to decrease serum total cholesterol, LDL cholesterol and triglycerides. The mechanisms behind these effects may be two fold. The reduction in cholesterol levels is likely due to an increase in bile acid synthesis. Andersson *et al.* [97] found that oat bran doubled the serum concentration of 7α-hydroxy-4-cholesten-3-one (α-HC), which is a metabolite in the synthesis of bile acids that is oxidized from 7α-hydroxycholesterol. The reduction in serum triglyceride levels may be a result of a decreased absorption of fat from the small intestine [95].

### 4.6. Cellulose

Cellulose is a linear chain of β(1→4) linked glucose monomers and is the structural component of cell walls in green plants and vegetables. It is water insoluble and inert to digestive enzymes in the small intestine. However, it can go through microbial fermentation to a certain degree in the large intestine in turn producing SCFA. 

Natural cellulose can be divided into two groups: Crystalline and amorphous. The crystalline component, which is made up of intra and intermolecular non covalent hydrogen bonds, make cellulose insoluble in water. However, many modified celluloses such as powdered cellulose, microcrystalline cellulose and hydroxypropylmethyl cellulose have been developed and are used as food ingredients. The difference between natural and modified celluloses is the extent of crystallization and hydrogen bonding. When these hydrogen bonds are disrupted and the crystallinity is lost, the cellulose derivative becomes water soluble [98].

Little research has been conducted evaluating the effects of cellulose in humans. Therefore, studies in other models such as the rat will be discussed. The translation to human relevance is poorly understood and debatable. Cellulose pills have been made available for human consumption with the theory that cellulose may decrease a person’s caloric intake. Although no human studies could be found to support this, several animal studies using cats [99], dogs [100] and rats [101] have shown that increasing dietary cellulose can reduce daily energy intake. This is most likely a dilution factor since cellulose is virtually undigested in the small intestine and only 51% metabolized by the microflora of the colon. 

Many studies have evaluated the effect of cellulose on blood glucose and insulin levels in many different models. However, the data is extremely contradictory and may depend on the subject, type of cellulose and other unknown factors. Using the rat [102], dog [103] and cat [104], natural cellulose was shown to decrease postprandial glucose and insulin levels. However, similar studies in pigs [105] and humans [106] demonstrated that natural cellulose had no effect on these parameters. Studies using modified celluloses showed more consistent data. Microcrystalline cellulose has shown the ability to decrease blood glucose levels in the pig [107] and rat [108]. Complimenting this, methylcellulose had demonstrated the same effects in humans. Lightowler and Henry [109] found that adding only 1% high viscosity hydroxypropylmethylcellulose (HV-HPMC) to mashed potatoes decreased postprandial glucose levels by 37% in healthy adults. Also, Maki *et al.* [110] reported an acute 35% reduction in postprandial blood glucose due to 4 g of HV-HPMC in overweight subjects. 

Modified cellulose has also been reported to effect lipid metabolism. Maki *et al.* [111,112] both observed a significant reduction in total and LDL cholesterol in hypercholesterolemic adults consuming 5 g/d of HV-HPMC for four weeks. Interestingly, in subjects already receiving statin drugs, HV-HPMC was able to further reduce total and LDL cholesterol. 

According to this, modified celluloses may be more beneficial than natural cellulose. These modified celluloses, as described above, act like soluble fiber thus adding to the viscosity of the GI tract. Therefore, it is assumed that increased intestinal viscosity delays nutrient absorption and increases bile acid excretion. 

### 4.7. Resistant Starch

Resistant starches (RS) are defined as any starch not digested in the small intestine [113]. RS behaves like soluble fiber without sacrificing palatability and mouth feel. Thus, resistant starch tries combining the health benefits of dietary fiber/whole grain with the sensory feel of refined carbohydrates. 

RS has been classified into four basic “types”. Type 1 (RS1) is made up of starch granules surrounded by an indigestible plant matrix. Type 2 (RS2) occurs in its natural form such as in an uncooked potato and high amylose maize. Type 3 (RS3) are crystallized starches made by unique cooking and cooling processes. Type 4 (RS4) is a starch chemically modified by esterification, crosslinking, or transglycosylation and is not found in nature. Few studies have compared types, but one recent study by Haub *et al.* [114] reported that cross-linked RS4 elicited a greater glucose lowering effect than the more commonly tested RS2. 

A majority of human studies involving RS have shown a decrease in postprandial blood glucose and insulin levels. However, it is difficult to completely understand these effects due to differences in study design and the type of RS used. Behall *et al.* [115] found that women consuming 0.71 g, 2.57 g or 5.06 g of RS had significantly lower postprandial glucose and insulin levels when compared to the control. However, this study failed to maintain an equal amount of available carbohydrate between the treatments and control. Therefore, it is difficult to determine whether the attenuation of glucose and insulin was due to the RS or the fact that there was less available carbohydrate in the meal. Similarly, Reader *et al.* [116] reported that 7.25 g of RS added to an energy bar decreased blood glucose and insulin levels in healthy adults. But, ingredients, amount of ingredients and nutrient levels were different for each treatment. A recent study by Al-Tamimi *et al.* [117], however removed these variables by controlling for non starch ingredients and available carbohydrates. It was reported that postprandial blood glucose and insulin levels were significantly reduced with the supplementation of 30 g of RS4.

Several studies report that longer term consumption of a RS may decrease fasting cholesterol and triglyceride levels. In a five week study, Behall *et al.* [118] found that men consuming 34% of their energy from high amylose maize, when compared to a high amylopectin carbohydrate, had significantly reduced fasting cholesterol and triglyceride levels. Resier *et al.* [119] reported similar results in an isocaloric and isonutrient diet with either high amylose maize or fructose. Porikos and Van Itallie [120] suggest that an interaction exists between sucrose, and therefore most likely fructose, and saturated fatty acids in turn promoting serum triglyceride levels. Interestingly, the relationship does not seem to exist for polyunsaturated fatty acids. The likely mechanism behind the ability of RS to decrease cholesterol levels is an increased intestinal viscosity. However, some studies, such as Jenkins *et al.* [121], report conflicting data as RS2 and RS3 had no effect on serum lipid profiles. While using the same type of RS, subjects were only tested for two weeks. It may be that the RS requires a longer period of time to promote an effect. 

Research has also been conducted which evaluates the effect of RS on fat oxidation and storage. However, data between studies are contradictory with no clear conclusions. Tagliabue *et al.* [122] reported that RS2, obtained from raw potatoes, was able to increase fat oxidation 5 h postprandial. However, the test diet, consisting of the RS2, had significantly less gross and metabolizable energy. Therefore, it is difficult to determine if the increased fat oxidation was due to the RS2 or a decreased caloric intake. A 10 week study by Howe *et al.* [123] may suggest the latter. High amylose starch, compared to a high amylopectin, produced no change on fat oxidation when an isocaloric diet was consumed. Conversely, Robertson *et al.* [124] reported that 30 g of RS2 added to healthy subjects habitual diet resulted in a significant decrease in subcutaneous abdominal adipose tissue non-esterfied fatty acid (NEFA) and glycerol release. This may be a result of increased peripheral SCFA metabolism or ghrelin secretions. 

## 5. Conclusions

In a simplified definition, dietary fiber is a carbohydrate that resists digestion and absorption and may or may not undergo microbial fermentation in the large intestine. This definition is essentially the basis to its correlation between consumption levels and possible health benefits. Dietary fiber consists of many different constituents, however; some are of particular interest and include arabinoxylan, inulin, β-glucan, pectin, bran and resistant starches. These individual components of dietary fiber have been shown to significantly play an important role in improving human health. Current research is paying particular attention to these elements; although further research is needed to better understand particular health claims and the mechanisms involved. 

A large amount of research has reported an inverse relationship between fiber consumption and the risk for coronary heart disease and several types of cancer. For that reason, the FDA has adopted and published the claim that increased consumption of dietary fiber can reduce the prevalence of coronary heart diseases and cancer. The mechanisms behind these findings are still unclear. However, it is thought to be attributed to several factors including increasing bile acid excretion, decreased caloric intake, increased short chain fatty acid production, carcinogen binding effects, increased antioxidants, and increased vitamins and minerals.

Although not as yet adopted by the FDA, dietary fiber is suggested to play a role in other conditions such as obesity and diabetes. Although some data are contradictory, a majority of studies regarding dietary fiber report a decrease of these two conditions with increased consumption of fiber. 

The digestive and viscosity characteristics of dietary fiber are the likely modes of action which affect diabetes and obesity risk. These mechanisms appear to decrease nutrient absorption, therefore, decreasing metabolizable energy. Dietary fiber may also be able to decrease gross energy of a food due to its lower energy density. 

Further studies are needed in certain areas of dietary fiber research. Those of particular interest are in the components of fiber such as β-glucan, arabinoxylan, resistant starches, *etc.* These sub fractions may give a better understanding of the health benefits of dietary fiber as well as the mechanisms behind them. 

## References

[1] Tucker L.A., Thomas K.S. (2009). Increasing total fiber intake reduces risk of weight and fat gains in women. J. Nutr..

[2] Meyer K.A., Kushi L.H., Jacobs D.R., Slavin J., Sellers T.A., Folsom A.R. (2000). Carbohydrates, dietary fiber, and incident type 2 diabetes in older women. Am. J. Clin. Nutr..

[3] Park Y., Brinton L.A., Subar A.F., Hollenbeck A., Schatzkin A. (2009). Dietary fiber intake and risk of breast cancer in postmenopausal women: The National Institutes of Health-AARP Diet and Health Study. Am. J. Clin. Nutr..

[4] Streppel M.T., Ocke M.C., Boshuizen H.C., Kok F.J., Kromhout D. (2008). Dietary fiber intake in relation to coronary heart disease and all-cause mortality over 40 y: The Zutphen Study. Am. J. Clin. Nutr..

[5] FDA (2008). Health claims: Fiber-contaning grain products, fruits and vegetables and cancer. Code of Federal Regulations.

[6] Nomura A.M., Hankin J.H., Henderson B.E., Wilkens L.R., Murphy S.P., Pike M.C., Le Marchand L., Stram D.O., Monroe K.R., Kolonel L.N. (2007). Dietary fiber and colorectal cancer risk: The multiethnic cohort study. Cancer Causes Control.

[7] Schatzkin A., Park Y., Leitzmann M.F., Hollenbeck A.R., Cross A.J. (2008). Prospective study of dietary fiber, whole grain foods, and small intestinal cancer. Gastroenterology.

[8] Young G.P., Hu Y., Le Leu R.K., Nyskohus L. (2005). Dietary fibre and colorectal cancer: A model for environment—gene interactions. Mol. Nutr. Food Res..

[9] Adlercreutz H., Hamalainen E., Gorbach S.L., Goldin B.R., Woods M.N., Brunson L.S., Dwyer J.T. (1987). Association of Diet and Sex-Hormones in Relation to Breast-Cancer. Eur. J. Cancer Clin. Oncol..

[10] FDA (2008). Health claims: fruits, vegetables, and grain products that contain fiber, particularly soluble fiber, and risk of coronary heart disease. Code of Federal Regulations.

[11] Pereira M.A., O’Reilly E., Augustsson K., Fraser G.E., Goldbourt U., Heitmann B.L., Hallmans G., Knekt P., Liu S.M., Pietinen P., Spiegelman D., Stevens J., Virtamo J., Willett W.C., Ascherio A. (2004). Dietary fiber and risk of coronary heart disease—A pooled analysis of cohort studies. Arch. Intern. Med..

[12] Story J.A., Furumoto E.J., Buhman K.K. (1997). Dietary fiber and bile acid metabolism—an update. Adv. Exp. Med. Biol..

[13] Amaral L., Morgan D., Stephen A.M., Whiting S. (1992). Effect of Propionate on Lipid-Metabolism in Healthy-Human Subjects. FASEB J..

[14] Esposito K., Nappo F., Giugliano F., Di Palo C., Ciotola M., Barbieri M., Paolisso G., Giugliano D. (2003). Meal modulation of circulating interleukin 18 and adiponectin concentrations in healthy subjects and in patients with type 2 diabetes mellitus. Am. J. Clin. Nutr..

[15] Ma Y.S., Griffith J.A., Chasan-Taber L., Olendzki B.C., Jackson E., Stanek E.J., Li W.J., Pagoto S.L., Hafner A.R., Ockene I.S. (2006). Association between dietary fiber and serum C-reactive protein. Am. J. Clin. Nutr..

[16] Ogden C.L., Carroll M.D., Curtin L.R., McDowell M.A., Tabak C.J., Flegal K.M. (2006). Prevalence of overweight and obesity in the United States, 1999–2004. J. Am. Med. Assoc..

[17] Mokdad A.H., Ford E.S., Bowman B.A., Dietz W.H., Vinicor F., Bales V.S., Marks J.S. (2003). Prevalence of obesity, diabetes, and obesity-related health risk factors, 2001. J. Am. Med. Assoc..

[18] Weickert M.O., Pfeiffer A.F.H. (2008). Metabolic effects of dietary and prevention of diabetes. J. Nutr..

[19] AAAC AACC adopts oat bran definition. http://www.aaccnet.org/news/pdfs/OatBran.pdf.

[20] DeVries J.W. (2003). On defining dietary fibre. Proc. Nutr. Soc..

[21] FAO/WHO Codex Alimentarius Commission http://www.codexalimentarius.net/download/standards/34/CXG_002e.pdf.

[22] Jones J. (2000). Update on defining dietary fiber. Cereal Foods World.

[23] Sizer F., Whitney E. (2008). Nutrition: Concepts and Controversies.

[24] Wong J.M., Jenkins D.J. (2007). Carbohydrate digestibility and metabolic effects. J. Nutr..

[25] Liu R.H. (2003). Health benefits of fruit and vegetables are from additive and synergistic combinations of phytochemicals. Am. J. Clin. Nutr..

[26] Weickert M.O., Pfeiffer A.F. (2008). Metabolic effects of dietary fiber consumption and prevention of diabetes. J. Nutr..

[27] Liu S., Stampfer M.J., Hu F.B., Giovannucci E., Rimm E., Manson J.E., Hennekens C.H., Willett W.C. (1999). Whole-grain consumption and risk of coronary heart disease: Results from the Nurses’ Health Study. Am. J. Clin. Nutr..

[28] Ferguson L.R., Chavan R.R., Harris P.J. (2001). Changing concepts of dietary fiber: Implications for carcinogenesis. Nutr. Cancer.

[29] Terry P., Giovannucci E., Michels K.B., Bergkvist L., Hansen H., Holmberg L., Wolk A. (2001). Fruit, vegetables, dietary fiber, and risk of colorectal cancer. J. Natl. Cancer Inst..

[30] Li Z., Bowerman S., Heber D. (2005). Health ramifications of the obesity epidemic. Surg. Clin. North Am..

[31] Koh-Banerjee P., Franz M.V., Sampson L., Liu S.M., Jacobs D.R., Spiegelman D., Willett W., Rimm E. (2004). Changes in whole-grain, bran, and cereal fiber consumption in relation to 8-y weight gain among men. Am. J. Clin. Nutr..

[32] Keenan H.A., Doria A., Aiello L.P., King G.L. (2006). Positivity of C-peptide, GADA and IA2 antibodies in type 1 diabetic patients with extreme duration. Diabetes.

[33] Baer D.J., Rumpler W.V., Miles C.W., Fahey G.C. (1997). Dietary fiber decreases the metabolizable energy content and nutrient digestibility of mixed diets fed to humans. J. Nutr..

[34] Isken F., Klaus S., Osterhoff M., Pfeiffer A.F.H., Weickert M.O. (2010). Effects of long-term soluble *vs.* insoluble dietary fiber intake on high-fat diet-induced obesity in C57BL/6J mice. J. Nutr. Biochem..

[35] Davidson M.H., McDonald A. (1998). Fiber: Forms and functions. Nutr. Res..

[36] Schneeman B.O. (1998). Dietary fiber and gastrointestinal function. Nutr. Res..

[37] Renteria-Flores J.A., Johnston L.J., Shurson G.C., Gallaher D.D. (2008). Effect of soluble and insoluble fiber on energy digestibility, nitrogen retention, and fiber digestibility of diets fed to gestating sows. J. Anim. Sci..

[38] Anderson J.W. (1985). Physiological and Metabolic Effects of Dietary Fiber. Fed. Proc..

[39] Keenan M.J., Zhou J., McCutcheon K.L., Raggio A.M., Bateman H.G., Todd E., Jones C.K., Tulley R.T., Melton S., Martin R.J., Hegsted M. (2006). Effects of resistant starch, a non–digestible fermentable fiber, on reducing body fat. Obesity (Silver Spring).

[40] Du H.D., van der A D.L., Boshuizen H.C., Forouhi N.G., Wareham N.J., Halkjaer J., Tjonneland A., Overvad K., Jakobsen M.U., Boeing H. (2010). Dietary fiber and subsequent changes in body weight and waist circumference in European men and women. Am. J. Clin. Nutr..

[41] Meyer K.A., Kushi L.H., Jacobs D.R., Slavin J., Sellers T.A., Folsom A.R. (2000). Carbohydrates, dietary fiber, and incident type 2 diabetes in older women. Am. J. Clin. Nutr..

[42] Hu F.B., Manson J.E., Stampfer M.J., Colditz G., Liu S., Solomon C.G., Willett W.C. (2001). Diet, lifestyle, and the risk of type 2 diabetes mellitus in women. N. Engl. J. Med..

[43] Schulze M.B., Liu S., Rimm E.B., Manson J.E., Willett W.C., Hu F.B. (2004). Glycemic index, glycemic load, and dietary fiber intake and incidence of type 2 diabetes in younger and middle–aged women. Am. J. Clin. Nutr..

[44] Jenkins D.J., Wolever T.M., Leeds A.R., Gassull M.A., Haisman P., Dilawari J., Goff D.V., Metz G.L., Alberti K.G. (1978). Dietary fibres, fibre analogues, and glucose tolerance: Importance of viscosity. Br. Med. J..

[45] Montonen J., Knekt P., Jarvinen R., Aromaa A., Reunanen A. (2003). Whole-grain and fiber intake and the incidence of type 2 diabetes. Am. J. Clin. Nutr..

[46] Salmeron J., Ascherio A., Rimm E.B., Colditz G.A., Spiegelman D., Jenkins D.J., Stampfer M.J., Wing A.L., Willett W.C. (1997). Dietary fiber, glycemic load, and risk of NIDDM in men. Diabetes Care.

[47] Weickert M.O., Mohlig M., Koebnick C., Holst J.J., Namsolleck P., Ristow M., Osterhoff M., Rochlitz H., Rudovich N., Spranger J., Pfeiffer A.F. (2005). Impact of cereal fibre on glucose–regulating factors. Diabetologia.

[48] Samra R., Anderson G.H. (2007). Insoluble cereal fiber reduces appetite and short-term food intake and glycemic response to food consumed 75 min later by healthy men. Am. J. Clin. Nutr..

[49] Brighenti F., Castellani G., Benini L., Casiraghi M.C., Leopardi E., Crovetti R., Testolin G. (1995). Effect of Neutralized and Native Vinegar on Blood-Glucose and Acetate Responses to a Mixed Meal in Healthy-Subjects. Eur. J. Clin. Nutr..

[50] Ostman E.M., Liljeberg Elmstahl H.G., Bjorck I.M. (2002). Barley bread containing lactic acid improves glucose tolerance at a subsequent meal in healthy men and women. J. Nutr..

[51] Ferrannini E., Barrett E.J., Bevilacqua S., DeFronzo R.A. (1983). Effect of fatty acids on glucose production and utilization in man. J. Clin. Invest..

[52] Crouse J.R., Gerson C.D., Decarli L., Lieber C.S. (1968). Role of Acetate in the Reduction of Plasma Free Fatty Acids Produced by Ethanol in Man. J. Lipid Res..

[53] Kelley D.E., Mandarino L.J. (2000). Fuel selection in human skeletal muscle in insulin resistance—A reexamination. Diabetes.

[54] Slavin J.L., Martini M.C., Jacobs D.R., Marquart L. (1999). Plausible mechanisms for the protectiveness of whole grains. Am. J. Clin. Nutr..

[55] Paolisso G., Barbagallo M. (1997). Hypertension, diabetes mellitus, and insulin resistance: The role of intracellular magnesium. Am. J. Hypertens..

[56] Jenkins D.J.A., Kendall C.W.C., Augustin L.S.A., Martini M.C., Axelsen M., Faulkner D., Vidgen E., Parker T., Lau H., Connelly P.W., Teitel J., Singer W., Vandenbroucke A.C., Leiter L.A., Josse R.G. (2002). Effect of wheat bran on glycemic control and risk factors for cardiovascular disease in type 2 diabetes. Diabetes Care.

[57] Ring S.G., Selvendran R.R. (1980). Isolation and Analysis of Cell-Wall Material from Beeswing Wheat Bran (Triticum-Aestivum). Phytochemistry.

[58] Lu Z.X., Walker K.Z., Muir J.G., Mascara T., O’Dea K. (2000). Arabinoxylan fiber, a byproduct of wheat flour processing, reduces the postprandial glucose response in normoglycemic subjects. Am. J. Clin. Nutr..

[59] Lu Z.X., Walker K.Z., Muir J.G., O’Dea K. (2004). Arabinoxylan fibre improves metabolic control in people with Type II diabetes. Eur. J. Clin. Nutr..

[60] Gibson G.R., Beatty E.R., Wang X., Cummings J.H. (1995). Selective stimulation of bifidobacteria in the human colon by oligofructose and inulin. Gastroenterology.

[61] Rafter J., Bennett M., Caderni G., Clune Y., Hughes R., Karlsson P.C., Klinder A., O’Riordan M., O’Sullivan G.C., Pool-Zobel B., Rechkemmer G., Roller M., Rowland I., Salvadori M., Thijs H., Van Loo J., Watzl B., Collins J.K. (2007). Dietary synbiotics reduce cancer risk factors in polypectomized and colon cancer patients. Am. J. Clin. Nutr..

[62] Griffin I.J., Hicks P.M.D., Heaney R.P., Abrams S.A. (2003). Enriched chicory inulin increases calcium absorption mainly in girls with lower calcium absorption. Nutr. Res..

[63] Abrams S.A., Griffin I.J., Hawthorne K.M., Liang L., Gunn S.K., Darlington G., Ellis K.J. (2005). A combination of prebiotic short- and long-chain inulin-type fructans enhances calcium absorption and bone mineralization in young adolescents. Am. J. Clin. Nutr..

[64] Cani P.D., Joly E., Horsmans Y., Delzenne N.M. (2006). Oligofructose promotes satiety in healthy human: A pilot study. Eur. J. Clin. Nutr..

[65] Wood P.J., Weisz J., Fedec P. (1991). Potential for Beta-Glucan Enrichment in Brans Derived from Oat (*Avena sativa* L.) Cultivars of Different (1→3),(1→4)-Beta-D-Glucan Concentrations. Cereal Chem..

[66] Doublier J.L., Wood P.J. (1995). Rheological Properties of Aqueous-Solutions of (1→3)(1→4)-Beta-D-Glucan from Oats (*Avena sativa* L.). Cereal Chem..

[67] Theuwissen E., Mensink R.P. (2007). Simultaneous intake of beta-glucan and plant stanol esters affects lipid metabolism in slightly hypercholesterolemic subjects. J. Nutr..

[68] Naumann E., van Rees A.B., Onning G., Oste R., Wydra M., Mensink R.P. (2006). beta-Glucan incorporated into a fruit drink effectively lowers serum LDL-cholesterol concentrations. Am. J. Clin. Nutr..

[69] Biorklund M., van Rees A., Mensink R.P., Onning G. (2005). Changes in serum lipids and postprandial glucose and insulin concentrations after consumption of beverages with beta‑glucans from oats or barley: A randomised dose-controlled trial. Eur. J. Clin. Nutr..

[70] Tappy L. (1995). Regulation of hepatic glucose production in healthy subjects and patients with non‑insulin-dependent diabetes mellitus. Diabete Metab..

[71] Lia A., Hallmans G., Sandberg A.S., Sundberg B., Aman P., Andersson H. (1995). Oat beta-glucan increases bile acid excretion and a fiber-rich barley fraction increases cholesterol excretion in ileostomy subjects. Am. J. Clin. Nutr..

[72] Nazare J.A., Normand S., Triantafyllou A.O., de la Perriere A.B., Desage M., Laville M. (2009). Modulation of the postprandial phase by beta-glucan in overweight subjects: Effects on glucose and insulin kinetics. Mol. Nutr. Food Res..

[73] Kim H.J., White P.J. (2010). *In Vitro* Bile-Acid Binding and Fermentation of High, Medium, and Low Molecular Weight beta-Glucan. J. Agric. Food Chem..

[74] Kim H.J., White P.J. (2009). *In Vitro* Fermentation of Oat Flours from Typical and High beta-Glucan Oat Lines. J. Agric. Food Chem..

[75] Ide T., Okamatsu H., Sugano M. (1978). Regulation by Dietary Fats of 3-Hydroxy-3-Methylglutaryl-Coenzyme-a Reductase in Rat-Liver. J. Nutr..

[76] Keogh G.F., Cooper G.J.S., Mulvey T.B., McArdle B.H., Coles G.D., Monro J.A., Poppitt S.D. (2003). Randomized controlled crossover study of the effect of a highly beta-glucan-enriched barley on cardiovascular disease risk factors in mildly hypercholesterolemic men. Am. J. Clin. Nutr..

[77] Cugnet-Anceau C., Nazare J.A., Biorklund M., Le Coquil E., Sassolas A., Sothier M., Holm J., Landin-Olsson M., Onning G., Laville M., Moulin P. (2010). A controlled study of consumption of beta-glucan-enriched soups for 2 months by type 2 diabetic free-living subjects. Br. J. Nutr..

[78] Suortti T., Johansson L., Autio K. Effect of heating and freezing on molecular weight of oat β–glucan. AACC Annual Meeting.

[79] Kerckhoffs D.A.J.M., Hornstra G., Mensink R.P. (2003). Cholesterol-lowering effect of beta-glucan from oat bran in mildly hypercholesterolemic subjects may decrease when beta-glucan is incorporated into bread and cookies. Am. J. Clin. Nutr..

[80] Yao N., Jannink J.L., White P.J. (2007). Molecular weight distribution of (1→3)(1→4)-beta-Glucan affects pasting properties of flour from oat lines with high and typical amounts of beta-glucan. Cereal Chem..

[81] Gaidosova A., Petruldkova Z., Havrlentova M., Cervena V., Hozova B., Sturdik E., Kogan G. (2007). The content of water-soluble and water-insoluble beta-D-glucans in selected oats and barley varieties. Carbohydr. Polym..

[82] Torronen R., Kansanen L., Uusitupa M., Hanninen O., Myllymaki O., Harkonen H., Malkki Y. (1992). Effects of an oat bran concentrate on serum lipids in free-living men with mild to moderate hypercholesterolaemia. Eur. J. Clin. Nutr..

[83] Cugnet-Anceau C., Nazare J.A., Biorklund M., Le Coquil E., Sassolas A., Sothier M., Holm J., Landin-Olsson M., Onning G., Laville M., Moulin P. (2010). A controlled study of consumption of beta-glucan-enriched soups for 2 months by type 2 diabetic free-living subjects. Br. J. Nutr..

[84] Braaten J.T., Wood P.J., Scott F.W., Wolynetz M.S., Lowe M.K., Bradley-White P., Collins M.W. (1994). Oat beta-glucan reduces blood cholesterol concentration in hypercholesterolemic subjects. Eur. J. Clin. Nutr..

[85] Lawaetz O., Blackburn A.M., Bloom S.R., Aritas Y., Ralphs D.N.L. (1983). Effect of Pectin on Gastric-Emptying and Gut Hormone-Release in the Dumping Syndrome. Scand. J. Gastroenterol..

[86] Brown L., Rosner B., Willett W.W., Sacks F.M. (1999). Cholesterol-lowering effects of dietary fiber: A meta-analysis. Am. J. Clin. Nutr..

[87] Jenkins D.J.A., Leeds A.R., Gassull M.A., Cochet B., Alberti K.G.M.M. (1977). Decrease in Postprandial Insulin and Glucose Concentrations by Guar and Pectin. Ann. Intern. Med..

[88] Rabbani G.H., Teka T., Zaman B., Majid N., Khatun M., Fuchs G.J. (2001). Clinical studies in persistent diarrhea: Dietary management with green banana or pectin in Bangladeshi children. Gastroenterology.

[89] Triplehorn C., Millard P.S. (2002). A rice-based diet with green banana or pectin reduced diarrhea in infants better than a rice-alone diet. ACP J. Club.

[90] Olano-Martin E., Gibson G.R., Rastell R.A. (2002). Comparison of the *in vitro* bifidogenic properties of pectins and pectic-oligosaccharides. J. Appl. Microbiol..

[91] Veldman F.J., Nair C.H., Vorster H.H., Vermaak W.J.H., Jerling J.C., Oosthuizen W., Venter C.S. (1999). Possible mechanisms through which dietary pectin influences fibrin network architecture in hypercholesterolaemic subjects. Thromb. Res..

[92] Nangia-Makker P., Hogan V., Honjo Y., Baccarini S., Tait L., Bresalier R., Raz A. (2002). Inhibition of human cancer cell growth and metastasis in nude mice by oral intake of modified citrus pectin. J. Natl. Cancer Inst..

[93] Ulmius M., Johansson A., Onning G. (2009). The influence of dietary fibre source and gender on the postprandial glucose and lipid response in healthy subjects. Eur. J. Nutr..

[94] Qureshi A.A., Sami S.A., Khan F.A. (2002). Effects of stabilized rice bran, its soluble and fiber fractions on blood glucose levels and serum lipid parameters in humans with diabetes mellitus Types I and II. J. Nutr. Biochem..

[95] Zhang J.X., Lundin E., Hallmans G., Adlercreutz H., Andersson H., Bosaeus I., Aman P., Stenling R., Dahlgren S. (1994). Effect of Rye Bran on Excretion of Bile-Acids, Cholesterol, Nitrogen, and Fat in Human-Subjects with Ileostomies. Am. J. Clin. Nutr..

[96] Jensen M.K., Koh-Banerjee P., Hu F.B., Franz M., Sampson L., Gronbaek M., Rimm E.B. (2004). Intakes of whole grains, bran, and germ and the risk of coronary heart disease in men. Am. J. Clin. Nutr..

[97] Andersson M., Ellegard L., Andersson H. (2002). Oat bran stimulates bile acid synthesis within 8 h as measured by 7 alpha-hydroxy-4-cholesten-3-one. Am. J. Clin. Nutr..

[98] Takahashi R., Hirasawa Y., Nichinari K. (2003). Cellulose and its derivatives. Foods Food Ingred. J. Jpn..

[99] Prola L., Dobenecker B., Kienzle E. (2006). Interaction between dietary cellulose content and food intake in cats. J. Nutr..

[100] Dobenecker B., Kienzle E. (1998). Interactions of cellulose content and diet composition with food intake and digestibility in dogs. J. Nutr..

[101] Delorme C.B., Wojcik J. (1982). Interaction of Dietary-Protein with Cellulose in the Adaptation to Caloric Dilution by Weanling Rats. J. Nutr..

[102] Schwartz S.E., Levine G.D. (1980). Effects of dietary fiber on intestinal glucose absorption and glucose tolerance in rats. Gastroenterology.

[103] Nelson R.W., Duesberg C.A., Ford S.L., Feldman E.C., Davenport D.J., Kiernan C., Neal L. (1998). Effect of dietary insoluble fiber on control of glycemia in dogs with naturally acquired diabetes mellitus. J. Am. Vet. Med. Assoc..

[104] Nelson R.W., Scott-Moncrieff J.C., Feldman E.C., DeVries-Concannon S.E., Kass P.H., Davenport D.J., Kiernan C.T., Neal L.A. (2000). Effect of dietary insoluble fiber on control of glycemia in cats with naturally acquired diabetes mellitus. J. Am. Vet. Med. Assoc..

[105] Nunes C.S., Malmlof K. (1992). Effects of guar gum and cellulose on glucose absorption, hormonal release and hepatic metabolism in the pig. Br. J. Nutr..

[106] Schwartz S.E., Levine R.A., Singh A., Scheidecker J.R., Track N.S. (1982). Sustained pectin ingestion delays gastric emptying. Gastroenterology.

[107] Low A.G., Pittman R.J., Elliott R.J. (1985). Gastric-Emptying of Barley Soybean Diets in the Pig—Effects of Feeding Level, Supplementary Maize Oil, Sucrose or Cellulose, and Water–Intake. Br. J. Nutr..

[108] Takahashi T., Karita S., Ogawa N., Goto M. (2005). Crystalline cellulose reduces plasma glucose concentrations and stimulates water absorption by increasing the digesta viscosity in rats. J. Nutr..

[109] Lightowler H.J., Henry C.J. (2009). Glycemic response of mashed potato containing high-viscocity hydroxypropylmethylcellulose. Nutr. Res..

[110] Maki K.C., Davidson M.H., Witchger M.S., Dicklin M.R., Subbaiah P.V. (2007). Effects of high‑fiber oat and wheat cereals on postprandial glucose and lipid responses in healthy men. Int. J. Vitam. Nutr. Res..

[111] Maki K.C., Davidson M.H., Torri S., Ingram K.A., O’Mullane J., Daggy B.P., Albrecht H.H. (2000). High-molecular-weight hydroxypropylmethylcellulose taken with or between meals is hypocholesterolemic in adult men. J. Nutr..

[112] Maki K.C., Carson M.L., Anderson W.H.K., Geohas J., Reeves M.S., Farmer M.V., Turowski M., Miller M., Kaden V.N., Dicklin M.R., Rains T.M. (2009). Lipid-altering effects of different formulations of hydroxypropylmethylcellulose. J. Clin. Lipidol..

[113] Higgins J.A. (2004). Resistant starch: Metabolic effects and potential health benefits. J. AOAC Int..

[114] Haub M.D., Hubach K.L., Al-Tamimi E.K. (2010). Different types of resistant starch elicit difference glucose responses in humans. J. Nutr. Metab..

[115] Behall K.M., Scholfield D.J., Hallfrisch J.G., Liljeberg-Elmstahl H.G.M. (2006). Consumption of both resistant starch and beta-glucan improves postprandial plasma glucose and insulin in women. Diabetes Care.

[116] Reader D.M., O’Connell B.S., Johnson M.L., Franz M. (2002). Glycemic and insulinemic response of subjects with type 2 diabetes after consumption of three energy bars. J. Am. Diet. Assoc..

[117] Al-Tamimi E.K., Sieb P.A., Snyder B., Haub M.D. (2010). Consumption of Cross-Linked Resistant Starch (RS4XL) on Glucose and Insulin Responses in Humans. J. Nutr. Metab..

[118] Behall K.M., Scholfield D.J., Yuhaniak I., Canary J. (1989). Diets Containing High Amylose vs Amylopectin Starch—Effects on Metabolic Variables in Human-Subjects. Am. J. Clin. Nutr..

[119] Reiser S., Powell A.S., Scholfield D.J., Panda P., Ellwood K.C., Canary J.J. (1989). Blood lipids, lipoproteins, apoproteins, and uric acid in men fed diets containing fructose or high-amylose cornstarch. Am. J. Clin. Nutr..

[120] Porikos K.P., Vanitallie T.B. (1983). Diet-Induced Changes in Serum Transaminase and Triglyceride Levels in Healthy Adult Men—Role of Sucrose and Excess Calories. Am. J. Med..

[121] Jenkins D.J.A., Vuksan V., Kendall C.W.C., Wursch P., Jeffcoat R., Waring S., Mehling C.C., Vidgen E., Augustin L.S.A., Wong E. (1998). Physiological effects of resistant starches on fecal bulk, short chain fatty acids, blood lipids and glycemic index. J. Am. Coll. Nutr..

[122] Tagliabue A., Raben A., Heijnen M.L., Deurenberg P., Pasquali E., Astrup A. (1995). The Effect of Raw Potato Starch on Energy-Expenditure and Substrate Oxidation. Am. J. Clin. Nutr..

[123] Howe J.C., Rumpler W.V., Behall K.M. (1996). Dietary starch composition and level of energy intake alter nutrient oxidation in “carbohydrate-sensitive” men. J. Nutr..

[124] Robertson M.D., Bickerton A.S., Dennis A.L., Vidal H., Frayn K.N. (2005). Insulin-sensitizing effects of dietary resistant starch and effects on skeletal muscle and adipose tissue metabolism. Am. J. Clin. Nutr..

